# A Central Council

**Published:** 1892-06-18

**Authors:** 


### PASSING TOPICS.

### A CENTRAL COUNCIL.

The *Charity Organization Review* of the current month contains a full report of the annual meeting of the Society, presided over by the Chairman of the Council, Mr. Timothy Holmes. That gentleman, in the course of his address to his constituents, waxed somewhat jubilant over the influence which, as he supposes, the C.O.S. has been able to bring to bear upon the hospital question, and he boldly claims the Lords' enquiry into the hospitals of London as "one of the branches of the Society's work."

We venture to think that this is a case in which a little modesty would not be out of place. It is, no doubt, perfectly true that the C.O.S. played an exceedingly astute part at the time the enquiry was in prospect, and managed to pose before the world as its virtual originator. But we take leave to doubt considerably whether Lord Sandhurst and his colleagues will be content to accept the humble position allotted to them by the spokesman of the Society, while we are equally certain that if they do they will merely invite the opposition of the hospitals to any proposals they are prepared to formulate.

The C.O.S. is regarded by the hospitals with a feeling stronger than suspicion. If there be one thing more than another upon which the hospitals may be said to be agreed, it is that they will not be placed under the control of an amateur body foreign to themselves, and when the Society talks of the part it has played in bringing about the anticipated interference with the affairs of institutions it has never been at any pains to assist, and which it has altogether failed to appreciate, it is surely reckoning without its host in assuming that the chief places at the Central Council it desires to see created will be allotted to its nominees. The hospitals will have none of them, and it remains to be seen how institutions which we are so often assured are voluntary in character and undoubtedly obtain the chief part of their incomes from voluntary sources, can be coerced into submission to an unacceptable authority.

Mr. Timothy Holmes, as a hospital surgeon, no doubt knows something of the opinions of certain of his fellow workers, but we doubt whether he has any right to adopt a representative attitude. Moreover, it is those who support the institutions who will have the last word. There is no question as yet, and we hope there never will be, between those who find the means and those who merely make use of those means, but it might soon arise in the absence of mutual respect and forbearance. Hospitals can never be maintained as voluntary institutions unless their philanthropic character is scrupulously guarded, and it would be difficult to pursuade most people that the practice of philanthropy is upon the programme either of the Charity Organization Society or of ardent medical economists.

The union of hospitals was proposed long before the Society began to meddle. But voluntary submission within certain limits to a board of accredited representatives is a very different thing from the acceptance of the dictation of an alien and wholly arbitrary and unsympathetic body, and the hospitals may surely be trusted to look to it that they are not caught napping.

Agricola.

